# The Foil for Filling Teeth

**Published:** 1894-02

**Authors:** H. L. Ambler

**Affiliations:** Cleveland, Ohio.


ARTICLE I.

### THE FOIL FOR FILLING TEETH.

BY DR. H. L. AMBLER, CLEVELAND, OHIO.

Read at World's Columbian Exposition.

When we take a retrospect and consider what a poor excuse tin foil was twenty or more years ago, we do not wonder that it failed so often to make tight, good-wearing fillings. When it came from the manufacturer it looked fairly bright, but after being exposed to the air it assumed a light brassy color, and lost what integrity it formerly possessed; No. 4 was generally used, but it would cut and crumble in the most provoking manner. At that time fillings were made by using mats, cylinders and hand pressure, similar to the use of non-cohesive gold, and it was very difficult to insert a respectable approximal filling. Several years ago a radical change came about, for which the manufacturers should have their share of credit; as with the tin we now have, you can begin at the base of any cavity and with mallet or hand force produce a filling which will be one compact mass from beginning to end, so that it can be cut and filed the same as gold, though not with so hard a surface as gold. Tin adheres, coheres, or welds, call it what you please, makes a saving solid, tight filling, and with less malleting than gold requires, for if over-malleted, the receiving surface is injured. It is a good material for filling many cavities in temporary teeth, and chlldren will bear having it used, because it can be done quickly and does not require much mallet or hand force to condense a single or double layer of No. 10. Up to the age of fourteen, or longer, we find many teeth which are quite chalky, with the oral fluids in such a condition that oxychlorid and oxyphosphate do not last long, and for some reason amalgam and gutta-percha soon fail. In all such cases we recommend tin, even in the incisors, for as the patient advances in years the tooth-structure generally becomes more dense, and if desirable the filling can be removed, and good saving operations made with gold. Approximal cavities in young teeth, filled with cohesive gold by good operators, often fail in one or two years, but refilled with tin the teeth are preserved. In approximal cavities attacked by white decay, the most formidable variety known, we would separate freely from the palatal or lingual side and fill carefully with tin.

It is invaluable when you are limited for time or means, and also for filling the first molars where we so often find poor calcification. Dr. S. B. Palmer says, "Tin not only arrests decay mechanically, but in frail, chalky structure acts as an anti-acid element in arresting the electric current set up between the tooth-structure and the filling-material." We *often* find the dentine beneath fillings which have been removed, considerably discolored, and greatly solidified as compared to its former condition, and we believe this condensation, or calcification, is more frequent under tin than gold. We have seen cases where the pulps had calcified under tin, and it has been known for years that tin would be tolerated in large cavities very near the pulp without causing any trouble. In many mouths tin does not oxidize, but retains a clean gray color. The objectionable color assumed where it does oxidize, is offset by the fact that the oxid fills the ends of the tubuli and often arrests further decay. Where fillings are subject to great attrition, they wear away sooner or later, but they can easily be replaced, and, as the portion against the walls is the last removed, further decay is prevented as long as there is any reasonable amount of tin left, and if the tooth-structure has become sufficiently solidified, you can cut proper anchorage and cover the tin completely with gold. It may be driven into or onto the tubuli, so as to completely close them from outside moisture, and often the tin takes such a hold that it requires a cutting instrument to remove it.

The extra tough foil now manufactured retains a bright surface, and does not lose its good qualities even after considerable exposure to the atmosphere, but we prefer to prepare only what is needed for each case, keeping the rest in the book placed in an envelope. Tin of this kind, well condensed by mallet or hand force, stays up against the walls of a cavity and makes a tight filling, and ought to be called perfect, because it preserves the tooth, probably expanding and contracting less than gold, and giving a surface which will wear from five to ten years, depending upon the size and location of the cavity. Buccal cavities in the first molars, and palatal cavities in the incisors, filled for children eight years old, are still in good condition after a period of fifteen years, and we have seen fillings twenty-five years of age. Strips of No. 10, from one to three thicknesses, can be welded together, cohering as well as semi-cohesive gold, or better, and can be manipulated much more rapidly; therefore, if desirable, you can produce any contour.

Some operators have advocated using gold and tin folded together in alternate layers, thus exposing both metals to the fluids of the mouth, claiming that fillings can be made quicker and are not as subject to thermal changes, and can be inserted nearer the pulp than when gold is used. These claims are entirely met by using tin alone. Others say that this union of gold and tin will preserve the teeth quite as well as a correct gold filling, but we do not see that it offers any advantage over either tin or gold, except that it wears somewhat longer than tin.

Instruments with square ends and sides, and medium serrations, are best adapted for hand force, and the majority of mediumly serrated hand-mallet instruments will work well on No. 10 tin of one, two, or three layers, using a four-ounce mallet with a fair, steady blow; but the force of blow will be guided by practice, thickness of tin, size of instrument, and depth of serrations. You must have absolute dryness and use care, not thinking because it is *tin* that it will be all right anyway. Fold the tin into strips of different widths, thickness, and lengths, according to size and location of cavity; but for a large crown or approximal cavity, the strips may be folded into mats or rolled into cylinders; but as more force is required to condense them, we generally prefer strips for frail teeth. To make the most *pliable* cylinders, cut a strip of any desired width from a sheet of foil and roll it on a broach, cutting it off to make cylinders of different thickness. When the cavity is full, go over the tin thoroughly with mallet or hand force, cutting down crown fillings with burs or corundum-wheels, and approximal fillings with sharp instruments, emery strips or discs. After partially finishing, give the filling another condensing with the burnisher, then a final trimming and moderate burnishing. By trimming fillings before they get wet, you can remedy any defects with a sharply-serrated plugger and thin strip of tin as easily as with gold.

Generally cavities are prepared the same as for gold, except that the grooves or pits should be a trifle larger. Many cavities can be filled with less excavating than required for gold, and some approximal cavities in bicuspids and molars can be well filled without removing the masticating surface. Here especially the cavities should be cut square into the teeth, so as not to leave a feather edge of tin when the filling is finished; but usually we cut the cervical margin down to a smooth strong edge, even if it goes beyond the gum or enamel margin. Now cut a *slight* groove across far enough from the margin so that it will not be broken out, make each end of this groove square or with *slight* pits, then from each pit cut a groove which will extend to the masticating surface. In nearly all approximal cavities in bicuspids and molars you will find some form of matrix of great advantage. By driving the tin firmly against the matrix you secure a well-condensed surface, and the teeth will move apart *slightly*, so that with a bevel or thin plugger you can force the tin between the matrix and the edge of the cavity, and thus be sure of having a tight filling, and plenty of material to finish well; then after removing the matrix condense with thin burnishers, and complete the finish as for gold. Where no matrix is used, or where it is used and removed before completing the filling, it is well to trim the cervical border, for in either case there is more light and room to work when only a portion of the cavity has been filled.

Be sure of all margins as you progress, and if the cavity is deep and a wide matrix shuts out the light, use a narrow one which can be moved toward the masticating surface as the work progresses. In the incisors and cuspids, where the labial or palatial wall is intact, this matrix can be bent at either end as the case requires, so as to make room for operating. We prefer to save the labial wall and line it with five layers of semi-cohesive gold folded into a mat and extended to the outer edge of the cavity; this gives the tooth a lighter shade. Bicuspids can be treated in the same way, a method originally used by Dr. Corydon Palmer. The tendency to crush or slide out during the process of filling is entirely overcome by using a matrix. We find that tin prevents further decay at the cervical margin of deep cavities oftener than any other metal or combination of metals. We fill from one-fourth to one-half of the cavity with tin, completing with gold when the tooth is of good structure, which gives all the advantages of gold for a masticating surface. Have the tin solid and square across the cavity, and the rest of the cavity of a good retaining form, the same as for a gold filling; then begin with a strip of gold slightly annealed and mallet it into the tin, but do not place too great reliance upon the connection of the two metals to keep the filling in place. We have sometimes filled incisors and cuspid approximal cavities along the labial margin with gold when the tooth was of medium structure. The fee should be reasonably large, for you can save many teeth for a longer time than with cohesive gold. Every good dentist with a little practice, can accomplish all that the writer claims to be done, as there is no special secret connected therewith.

### DISCUSSION.

Dr. E. T. Darby.—The author of this paper has certainly paid a very high, and I don't know but a very worthy, tribute to tin. There is nothing new in the paper, as far as I know; the use of tin almost antedates the use of gold as a filling material. The French first used lead, then tin; tin was used as early as 1800 in this country, to the exclusion of almost every other materal. In the earlier years of my practice, tin foil was used a good deal, and amalgam very little; there was a prejudice in the minds of many practitioners against the use of amalgam, because it was composed of equal parts of silver and tin combined with mercury. Consequently, the chief metal used was tin. Soon after I began to practice for myself, some one suggested to me a good method for preparing tin. Make an ordinary sand mould, and then melt chemically pure block tin in a spoon and pour it into this mould, making the tin in the form of a corundum-wheel. This is to put on a lathe, then with a very sharp chisel the tin is turned off, making shavings as thin or thick as desired. They can be made exceedingly thin, and are exceedingly tough.

During my college days, some members of the Faculty said tin would weld, and others said it would not. I took a tooth to my room, cut the crown off, and invested the roots with plaster of Paris, restoring the whole crown to its natural size with tin foil. I polished it up nicely, then took it to one of the professors who questioned the cohesive properties of tin, and said to him, "This tooth has been built up with ropes of tin on two retaining points." He said, "You have melted that tin." I said,

"No, sir, I built that up in my room under the eyes of some of my college mates, and it has been done as I said." He expressed surprise. I have kept that tooth, and show it every year as one of the evidences of the cohesive properties of tin.

I have always said that tin was one of the very best filling materials we have. I believe more teeth could be saved with tin than with gold. Whether tin possesses the antiseptic properties in as great a degree as is claimed by many, I sometimes question, but I do know that tin has a saving quality that we do not always find in gold. The method of combining tin and gold is not used. Dr. Jenkins, of Dresden, was the first advocate of filling teeth with tin and gold. I have been in the habit of combining tin and gold in some cavities, but I do not see any especial advantage in it. I cannot see that the filling is any better by incorporating the gold with the tin. There is but one disadvantage that tin possesses, so far as I am aware, that is its color, but in all approximal cavities that are exposed to view, I believe the average dentist will do as well with tin as with gold. I believe if the dental profession would use more tin, they would save more teeth. For children's teeth I know of nothing better for masticating surfaces. A good tin filling will condense upon the masticating surfaces of children's teeth, and, I think, save them better than anything else. I should use it much oftener than I do if it were not unsightly in the mouth. I endorse heartily and emphatically the tribute which the essayist has paid to tin.

Madam Tiburtius Hirschfield (of Vienna) heartily endorsed the use of tin and gold, after a practice with this material for twenty-four years. Like Dr. Darby, she tested the cohesive properties of tin, by building up some crowns with this material. Her practice had been mainly for children and ladies, and she thinks for filling children's teeth there is no better material than tin and gold. Sometimes these fillings were put in when the child was seven or eight years old, and at the age of seventeen the fillings were still perfect. She makes a filling of tin and gold that looks nearly as yellow as gold. For this filling, two sheets of gold No 4, and one of tin, *very thin*, are used. It looks just as bright after having been worn two years, as when it was first put in.

Dr. R. R. Freeman, (Nashville, Tenn.)—You don't know how happy I feel to hear the subject of tin foil brought up before this Congress. I learned something of tin in the early school, when I had the honor to be upon the stage with Madam Hirschfield, when we received our diplomas. I have written upon tin foil; I have talked upon it and advocated its use. I remember Dr. Truman said that tin foil was one of the best fillings, not excepting gold. I know what it is doing, and I know what it has done for twenty-five years.

All through our southern country we have those who are using tin foil for its therapeutic properties; it has that healing property for the dentine of children's teeth that hardens them, and it has been only a few years ago that one of our best practitioners said, "I must acknowledge that tin does retain teeth." A very wealthy family was once summering with me, and while there, their teeth needed some attention. Some of the teeth looked dark, and I found that the teeth were decaying. It was with considerable effort that I tried to save those teeth; and their dentist came to me with the enquiry why I had filled teeth for patients with tin, who were abundantly able to pay for gold. I told him, in order that the teeth might become developed and hardened under the tin, and that it did so was evident by the trouble he had in trying to cut away the tin fillings. I remember reading an article by Dr. Chase, of St. Louis, in 1869, advocating the placing of tin over sensitive dentine, in order to secure a gentle galvanic action—the galvanic action was said to be therapeutic and hardened the teeth. I remember a case in which I tried it. I made a tin disc and adjusted it very nicely over the soft dentine, and proceeded to fill the tooth with gold. After removing the rubber dam, I found the tin had slipped out to the margin and made a form like a crescent. It was for one of a family whose teeth were exceedingly soft. This was a very bad tooth, and was very sensitive at the time. I called the patient's attention to the fact, and watched that tooth with considerable care, and after three or four years I had to renew the fillings of teeth that had not been so lined, but this tooth was in a perfect condition, and it stands to-day since 1869, a perfect tooth at the cervical margin.

I am glad to give my testimony in behalf of tin foil.

Dr. Albert H. Brockway (Brooklyn, N. Y.)—I am especially pleased with this paper, but we must not forget we have a great variety of cavities to fill, in a great variety of situations, in a great variety of teeth of different characters; so that, if you are to do the best thing for the patient, be extremely eclectic in practice. It is for us to determine what to use for a given case under given conditions. I am a strong believer in the use of tin foil in such cases as will admit of it. I use it more or less, and I use it for two reasons especially. The first is from its adaptability and facility with which a saving filling can be made in favorable cases. I am also inclined strongly to believe in its therapeutic properties. I am not so sure of this, but experience seems to bear it out. In soft, chalky teeth, where the conditions are not favorable for tin, we have to resort to other materials. I use it especially in children's teeth, in cases where tin foil has been strongly recommended, and in which recommendation I quite agree; but there are many cases in children's teeth where it seems to me that tin foil could not be used so successfully as other materials, notably gutta-percha.

I wished simply to speak of the limitations of the usefulness of tin foil.

Dr. Gordon White (Nashville, Tenn.)—I claim for tin, after having used it for nine years, that it is the best filling material that has yet been given our profession, excepting that it will not stand friction. I think it is the best tooth-preserver that we have. When I first used it I combined it with gold, and I found that the two foils worked very harshly in my hands. After using it a couple of years that way, I commenced to use the foils separately, covering the tin with gold. I find it works very much softer when the foils are not introduced separately.

Dr. C. S. Stockton (Newark, N. J.)—It is not necessary for me to go over the ground so well covered by Dr. Darby, but there are people who come to me who are unable to pay the large fees that are requisite where you would use gold. I recall two fillings that I saw only a short time since, which were put in twenty-three years ago, I think. I filled those teeth by a plan recommended by Dr. Palmer, using mats of Abbey's soft foil packed up against the labial surfaces of the enamel, filling the balance of the teeth with tin foil. They are in as good condition to-day as they were twenty-three years ago. It is necessary and right to save the teeth of those who are not able to pay the large expense of gold work, and if we have a material that will save teeth, it seems to me it is our duty to use it. Tin is one of the best materials for saving teeth, and we should use it more than we do.

Dr. St. George Elliott (London, Eng.)—It is to me a very great pleasure to return to this country and find that tin is at least beginning to have a large number of advocates. In Europe we all look upon Dr. Abbott, in Berlin, as the father of modern dentistry there. He was one of the earliest, though not the first, to use tin foil, and he did so very successfully. He carried it out in his own practice, and his son-in-law, Dr. Miller, took it up, as did also Dr. Jenkins, of Dresden. For ten years I have used it very largely. I have averaged from four to five or six fillings a day. The greatest advantage of tin and gold has not been spoken of. You know if you get a preparation of tin and gold in correct proportion, there is practically a chemical union between the two, and you get not only hardness, but a certain amount of expansion. It is exceedingly valuable in filling crown cavities of molars. Its hardness is not immediately gotten. It takes from one to three years to harden. Its color is its advantage; it approaches that of amalgam. If you will use certain proportions you get a better color, but you get it at the expense of hardness.

Prof. James Truman (Philadelphia, Pa.)—It is with a great deal of gratification that I find, even at this late day, after forty years of practice in the use of tin foil, that it is coming up again with honor. I have long been satisfied that the profession had lost much in the abandonment of this material; perhaps the reason has been largely due to the fact that tin foil originally, as my friend Dr. Darby said, was not well made; then again, less attention was paid to having the sufaces clean, and also from the fact that very few who practiced with this material used it as I would, that is strictly upon the cohesive principle. In using the tin foil on the soft gold plan, it is necessary as far as my observation goes that the foil be packed in the cavity as solidly as gold. When this is accomplished, you have a filling that will resist mastication upon any surface. I have tested this for years, and I find that masticating surfaces filled with this character of material last from twenty to twenty-five years perfectly. The therapeutic properties of tin foil have been spoken of, and I believe there is a chemical action on the tooth-structure, but what that is I am not prepared to say. You will find tin foil much better adapted for use in the case of soft teeth. It has been asserted that it is better for children's teeth, but I would place it in all teeth except the anterior teeth, they can be better filled with it where the teeth are of soft character than with any other filling. Instead of using so much amalgam as we do, take tin, and it will be found more useful than gold in many respects. I agree with my friend in regard to its use at the cervical border. I remember in Dresden, in Dr. Jenkin's office he said to me, "I cannot, as some other men do, preserve the cervical border of teeth with gold, and therefore I invariably use tin and gold."

One word in regard to tin and gold. I have used it a good deal, and have seen Dr. Abbot operate with it in Berlin, and I know a good deal about Dr. Miller's use of it, and I am satisfied that it is a most valuable combination. It can be placed in wet, and if it is placed in wet it is better than when dry, owing to the action of the fluids of the mouth producing galvanic action between the two metals, that produces hardness. I remember once I had occasion to remove an anterior approximal filling of Dr. Abbott's, and I found that the tin and gold was as hard as any amalgam filling I ever saw, and I had great difficulty in cutting it out. That was due to galvanic action between the two metals. I feel gratified that this matter has come up this afternoon.

Dr. Jarvie, Chairman.—A question is asked if tin is cohesive under water.

Dr. Truman.—Not very well, but I have filled cavities under water. If you make a filling from shavings, you get the most cohesive property possible.

Dr. A. W. Freeman (Chicago, Ill.)—None have yet spoken of finishing tin foil with gold, that I remember. I fill approximal cavities nearly full or three-fourths full oftentimes with tin and gold or tin alone, and then finish with gold, using sometimes first a little soft gold and then finishing with cohesive gold. If you have your masticating surfaces carefully prepared, and if you are careful about some little undercuts, you can very often make a filling that will be just as durable as any gold filling. I have been surprised in looking back over my experience for eight or nine years to see how tin and gold, finished with gold, has preserved the teeth. The first cases I remember to have had my attention drawn to were by Dr. Allport. He said he used cohesive gold on the outside; now we use more tin and less gold, but always wrap the tin on the inside of the gold.

I endorse the use of tin and gold, and especially do I believe in its chemical action.—*Dominion Dental Journal.*

